# Successful treatment with secukinumab in a 4-month-old infant with severe generalized pustular psoriasis: a case report

**DOI:** 10.3389/fmed.2025.1713628

**Published:** 2025-11-14

**Authors:** Long Wang, Yirong Li, Chen Li, Lizhen Zhang, Lequ Zeng

**Affiliations:** 1 Department of Dermatology and Venereology, Zhongshan City People's Hospital, Zhongshan, Guangdong, China; 2 The First Clinical Medical College, Guangdong Medical University, Zhanjiang, Guangdong, China

**Keywords:** generalized pustular psoriasis, infant, secukinumab, efficacy and safety, case report

## Abstract

**Introduction:**

Secukinumab has demonstrated favorable efficacy and safety in the management of plaque psoriasis; however, its use in generalized pustular psoriasis (GPP) remains limited, particularly in children under 6 years of age, with only a few clinical reports available.

**Case description:**

A 4-month-old infant was admitted with a 2-month history of GPP, which had worsened over the previous week with extensive erythematous rashes and pruritus. More than 70% of the body surface area was affected, with progression toward erythroderma. The Japanese Dermatological Association Severity Index score was 7, indicating moderate severity. Treatment with secukinumab 50 mg weekly, combined with daily emollients, was initiated. Complete clearance of skin lesions was achieved after four injections. One year after discontinuation of secukinumab, recurrence occurred; a single re-administration of secukinumab resulted in near-complete resolution.

**Conclusion:**

Secukinumab may provide potential clinical benefits with an acceptable safety profile in infants with GPP. Further research is required to establish the optimal treatment duration, dosing intervals, and long-term safety in this population.

## Introduction

Generalized pustular psoriasis (GPP) is a rare and severe psoriasis subtype characterized by widespread sterile pustules, often accompanied by systemic manifestations such as fever, arthralgia, and leukocytosis (1). Recurrent and cyclic flares are common, and in severe cases, delayed treatment may lead to secondary infection or even organ failure, highlighting the need for timely medical intervention (1). Secukinumab, a fully human monoclonal antibody targeting interleukin-17A, has demonstrated favorable efficacy and safety in plaque psoriasis (2). However, its use in GPP remains uncommon, particularly in pediatric patients under 6 years of age, with only limited clinical evidence available. The present case describes a 4-month-old infant with severe GPP who achieved complete resolution following treatment with secukinumab.

## Case description

A 4-month-old infant was admitted to Zhongshan People's Hospital for a 2-month history of GPP that had aggravated with rashes and itching in recent 1 week. Two months ago, the patient developed generalized erythema without significant desquamation, vesicles, or pustules. She was initially diagnosed with eczema at a local hospital. She was treated with Lianbo Qushi Zhiyang lotion (a traditional Chinese medicine preparation), desonide cream for topical use, and loratadine syrup orally. However, her symptoms did not improve. One week before admission, multiple millet-sized pustules appeared on the background of existing erythema.

**Figure 1:**
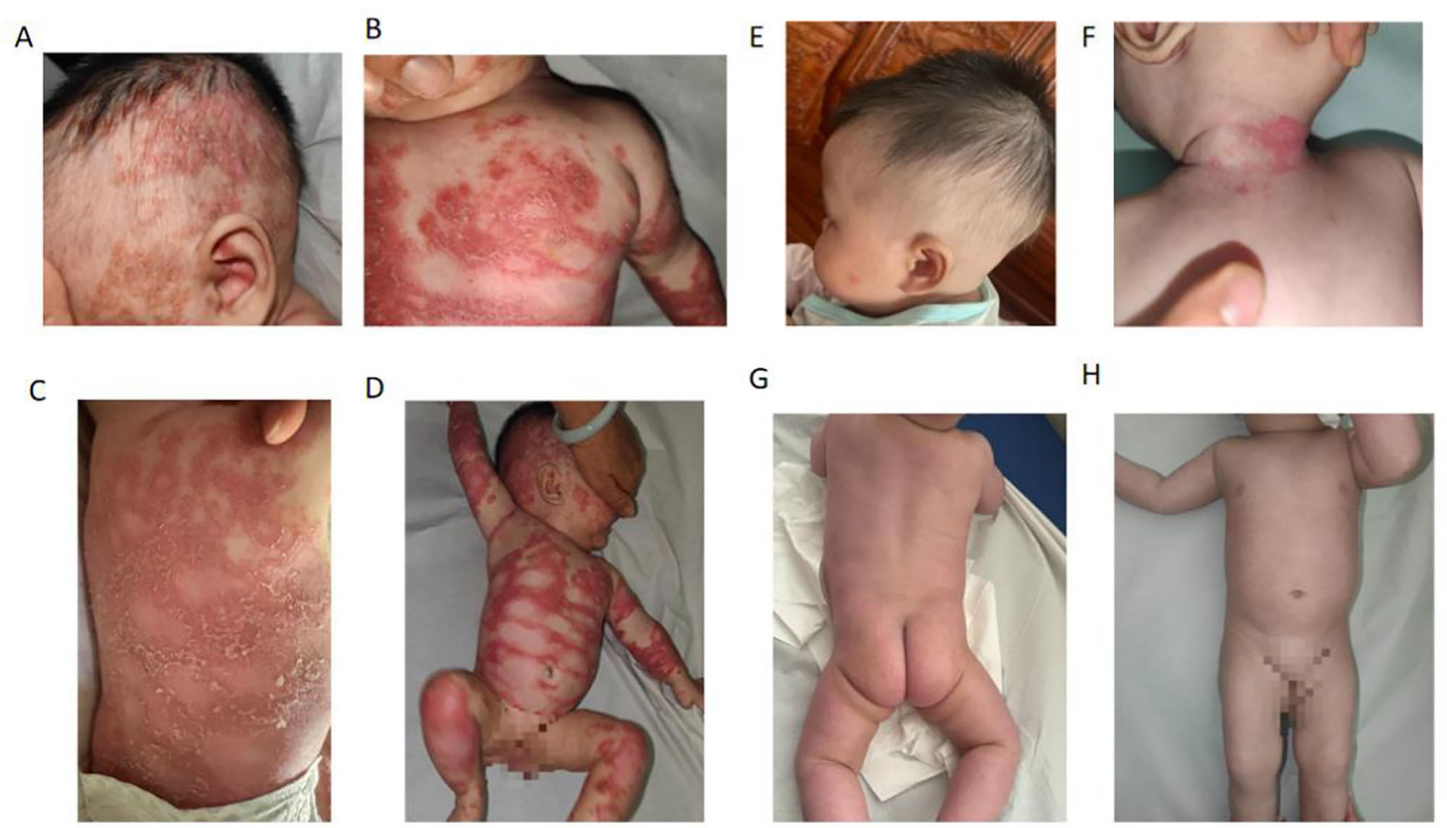
Generalized pustular psoriasis (GPP) in a 4-month-old female before **(A–D)** and after **(E–H)** treatment. **(A–D)** A diffuse erythema covered with confluent pustules all over the trunk and limbs, leading to the formation of a pustular lake with superficial scaling at a later stage. **(E–H)** The patient was treated with a 50-mg dose of secukinumab once a week and daily application of emollients. The rashes resolved rapidly within 4 weeks.

The patient's mother has a history of psoriasis with mild skin involvement and has not received regular treatment. At physical examination, the patient showed widespread erythema of varying sizes over the entire body. On the trunk and limbs, the erythematous lesions had largely coalesced into confluent plaques. Numerous pinhead-sized pustules were densely distributed along the margins of the erythema throughout the body. On the trunk, these pustules had merged to form lakes of pus. Some of the erythematous areas showed mild edema. The affected skin area exceeded 70% of the total body surface area, showing a tendency toward erythroderma (Figures 1A–D). No erosions or ulcers were observed in the oral cavity or genital area. Hair, fingernails, and toenails appeared normal.

The pustular lesions were assessed using the Japanese Dermatological Association Severity Index (JDA-SI). The JDA-SI includes the evaluation of cutaneous symptoms (erythema, pustules, and edema; each scored from 0 to 3, total of 0–9 points) and systemic inflammation (fever, white blood cell count, C-reactive protein, and albumin; each scored from 0 to 2, total of 0–8 points). The combined score ranges from mild (0–6) to moderate (7–10) and severe (11–17). This infant was assigned a JDA-SI score of 7 points based on erythema, pustules, and edema, consistent with moderate disease severity.

**Figure 2:**
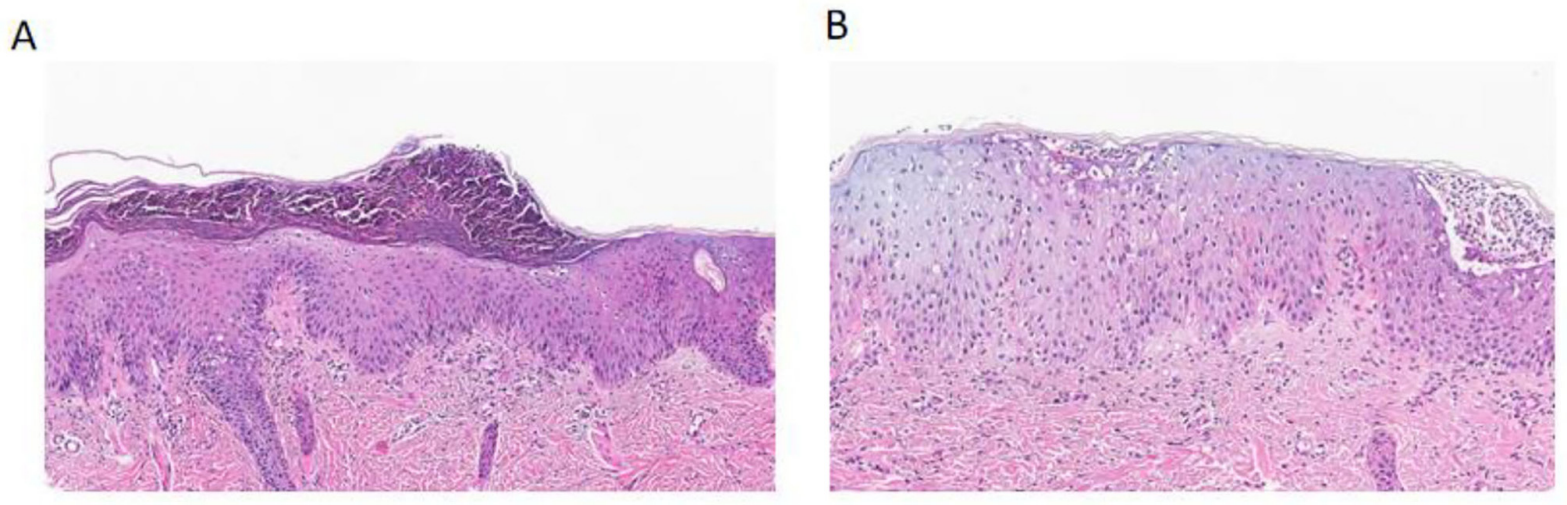
Histopathological findings of generalized pustular psoriasis compared with normal skin. **(A)** Hematoxylin and eosin (H&E) staining of the patient's lesional skin shows marked epidermal hyperplasia with keratosis and hypogranulosis, thinning of the granular layer, and thickening of the basal layer. Numerous neutrophils are observed within the stratum corneum and above the spinous layer, forming Munro's microabscesses, consistent with generalized pustular psoriasis. **(B)** H&E staining of normal skin demonstrates a well-preserved epidermal architecture without keratosis, hypogranulosis, or neutrophilic infiltration.

A skin biopsy was performed in the outpatient clinic, and confirmed the diagnosis of GPP (Figure 2). The epidermal hyperplasia was accompanied by keratosis and hypogranulosis. The granular layer was reduced, and the basal layer was thickened. Additionally, a significant presence of neutrophils was observed in the superficial keratosis and above the spinous layer. Genetic testing revealed a positive mutation in the CARD14 gene in the patient. A specific missense mutation, c.2458C>T, was identified, leading to a substitution of arginine for tryptophan at codon 820 (p.R820W). The report also noted other silent mutations in the CARD14 gene, including a synonymous mutation (c.633G>A, p.E211=) and several intronic and non-coding exonic variants, which are not expected to affect protein function (Supplementary Appendix 1). The parents declined genetic testing for themselves for economic reasons (no medical insurance).

Pesolizumab was first considered for treatment, but its cost in mainland China is about 34 times higher than secukinumab, and the patient had no commercial medical insurance. Therefore, the patient was treated with a 50-mg dose of secukinumab once a week and daily application of emollients. The recommended dosing regimen for secukinumab is 75 mg for patients weighing < 25 or 25–50 kg, 150 mg for patients weighing 25–50 or ≥50 kg, and 300 mg for patients weighing ≥50 kg. All doses are administered subcutaneously at weeks 0, 1, 2, 3, and 4, followed by administration every 4 weeks thereafter. Given that the patient was only 4 months old and the treatment constituted an off-label use, she was administered secukinumab 50 mg subcutaneously once weekly (qw). After four injections, the patient's skin lesions had completely resolved (Figures 1E–H).

**Figure 3:**
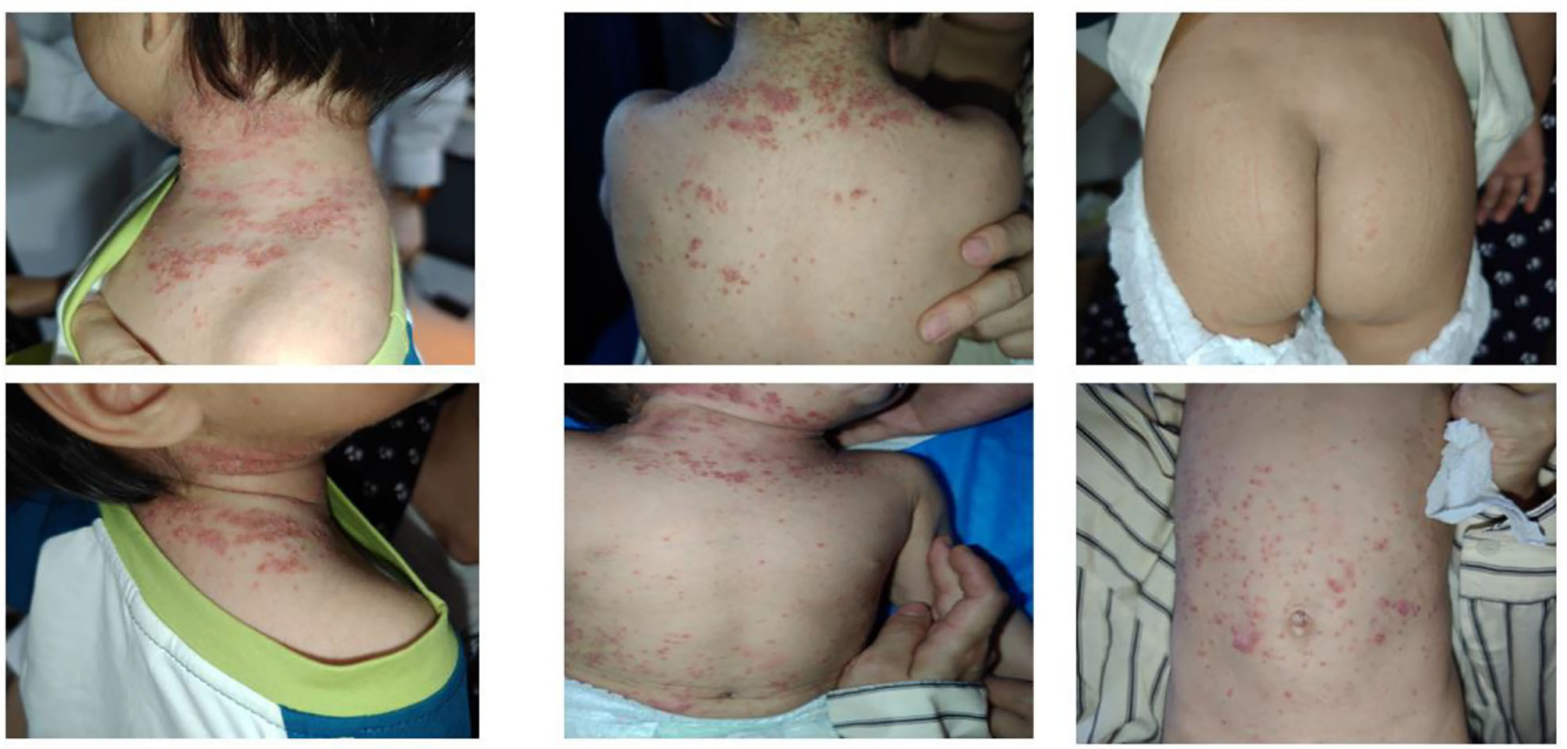
Recurrence observed at follow-up on April 22, 2025.

**Figure 4:**
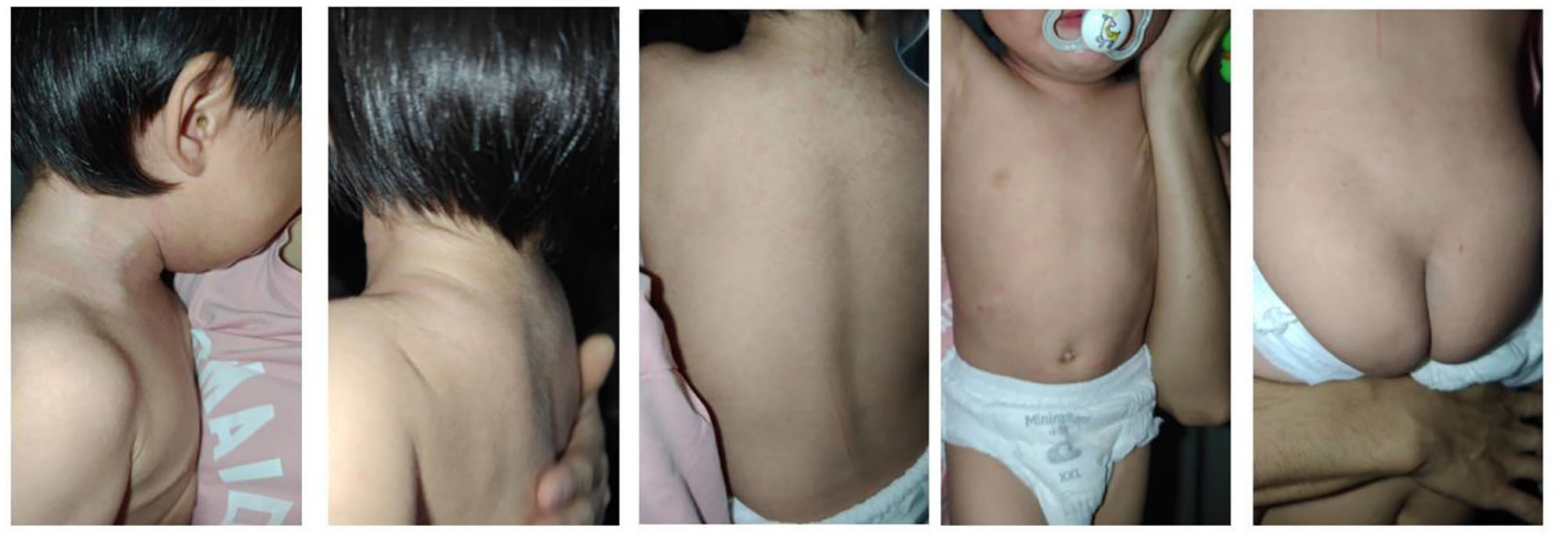
Follow-up visit on May 13, 2025.

The patient experienced a recurrence of skin lesions 1 year after discontinuation of secukinumab (Figure 3). In April of this year, she was retreated with secukinumab 75 mg subcutaneously once weekly (qw). On follow-up visit on May 13, the skin lesions were observed to have fully disappeared (Figure 4).

## Discussion

Secukinumab has demonstrated favorable efficacy and safety in the treatment of plaque psoriasis. Still, it is rarely used for pustular psoriasis, and there are a few clinical reports of secukinumab for psoriasis in children under 6 years old with GPP. The case reported here suggests that secukinumab may have clinical benefits in infants with GPP.

The management of GPP remains inadequate, partly due to the rarity of GPP (3). Avitrin, cyclosporine, and methotrexate are considered the preferred medications for acute GPP. Therefore, long-term treatment is usually necessary to minimize recurrence (1). Due to the limited availability of high-quality data on the efficacy of GPP treatment, the optimal treatment approach remains uncertain (1). In recent years, biologics targeting IL-12/23p40, IL-17A, and IL-36 have been reported for the treatment of GPP. Recent studies suggest that secukinumab can rapidly improve inflammatory markers in patients with GPP (4–8). Still, none of these reports included a patient as young as 4 months. Although no conclusion can be drawn from a single case, the present study suggests that it may be possible to treat infants with secukinumab. Of course, such a use should be reserved for moderate-to-severe cases, such as the one reported here, with lesions covering 70% of the body surface. Treatments should be performed as soon as possible in such young patients to avoid long-term complications. Of course, classical drugs for GPP, such as cyclosporine and methotrexate, are contraindicated in infants. For children younger than 6 years with plaque psoriasis, 4 years with enthesitis-related arthritis, and 2 years with psoriatic arthritis, the safety and efficacy of secukinumab have not been established, and its use is not recommended (9). If a clinical scenario involves a pediatric specialist, they should direct an infant's therapy, which is likely to involve alternative treatments. The use of secukinumab should be discussed in the team and with pharmacovigilance committees to ensure that no other options are available.

GPP is a severe and uncommon variant of psoriasis characterized by widespread pustule formation, fever, and systemic inflammation. Rapid symptom control is crucial, especially in pediatric patients. GPP exhibits high expression of IL-17A, IL-36, TNF-α, and neutrophil-attracting chemokines. IL-17A is particularly potent in driving neutrophil migration to the epidermis, an essential mechanism for pustule development (10, 11). IL-23 is upstream of IL-17A; it promotes the differentiation and survival of Th17 cells, which then produce IL-17A. However, in GPP, increased IL-36 activity uniquely amplifies IL-17A signaling and neutrophil infiltration, making IL-17 a more direct target (11). IL-17 inhibitors (e.g., secukinumab) show more rapid and pronounced clearance of pustules, fever reduction, and systemic symptom relief compared to IL-23 inhibitors (10, 12, 13). Studies demonstrated significantly higher rates of PASI 90/100 responses with IL-17 inhibitors at week 12 (PASI 90: IL-17 54% vs. IL-23 19%; PASI 100: IL-17 40% vs. IL-23 6%). At week 24, more patients on IL-17 inhibition had complete responses (IL-17 74% vs. IL-23 25%) (10). Secukinumab in GPP led to complete pustule clearance within 72 h for most, while IL-23 inhibitors (e.g., ustekinumab) took a week (11, 13). Some mechanistic reasons may support the superiority of IL-17 inhibition over IL-23 inhibition. IL-17A activates keratinocytes to release CXCL1, CXCL8, and other neutrophil chemoattractants more potently than IL-23. This drives the hallmark neutrophilic infiltrate seen in pustular lesions (11). IL-17 inhibitors directly block this signal, preventing neutrophil accumulation and pustule formation, whereas IL-23 inhibitors act upstream and may not fully suppress IL-17A activity (10, 14). IL-36 (a driver in GPP) acts in synergy with IL-17A rather than IL-23 (10). Hence, those reasons may explain the rapid onset and greater efficacy of IL-17 inhibition in pustular cases. Some IL-17 inhibitors are available. Secukinumab targets IL-17A. It demonstrates rapid onset, high PASI response rates, and sustained efficacy in GPP, including in infants and pediatric cases (7, 11, 12). Ixekizumab is also an IL-17A inhibitor; some studies suggest it offers similar or slightly improved long-term efficacy and drug survival compared to secukinumab, though tolerability is slightly lower (14, 15). Brodalumab targets the IL-17 receptor A, thus blocking both IL-17A and IL-17F. It may offer broader blockade and effective skin clearance, but has unique safety considerations (e.g., depression risk) (16). Therefore, IL-17 inhibitors, particularly secukinumab, appear to provide superior and fast control of pustular psoriasis by targeting the final common pathway for neutrophil recruitment and pustule formation. IL-23 inhibitors, acting upstream, do not fully suppress this process and show slower, less complete responses. Nevertheless, treatment affordability (e.g., in the case of pesolizumab) or availability (e.g., brodalumab is not available everywhere in China) by the patients should be considered.

Since the patient was very young, although the literature suggests that a 75-mg dose could be used, the authors had no prior clinical experience with biologic therapy for infantile GPP. Therefore, to ensure safety, a 50-mg dose was administered. The subsequent clinical outcome showed that the lesion clearance rate with the 50-mg dose was highly satisfactory.

This case suggests that secukinumab could be a promising therapeutic option for infants with GPP. However, further research is needed to determine when to discontinue secukinumab and whether infants need to be dosed at intervals during treatment. More clinical data is required to support this conclusion.

## Data Availability

The original contributions presented in the study are included in the article/Supplementary material, further inquiries can be directed to the corresponding author.
